# Increased astrocyte expression of IL-6 or CCL2 in transgenic mice alters levels of hippocampal and cerebellar proteins

**DOI:** 10.3389/fncel.2014.00234

**Published:** 2014-08-14

**Authors:** Donna L. Gruol, Khanh Vo, Jennifer G. Bray

**Affiliations:** ^1^Molecular and Cellular Neuroscience Department, The Scripps Research InstituteLa Jolla, CA, USA; ^2^Department of Biology, University of Wisconsin-Stevens PointStevens Point, WI, USA

**Keywords:** neuroimmune, western blot, synapse, signal transduction, GFAP, STAT3

## Abstract

Emerging research has identified that neuroimmune factors are produced by cells of the central nervous system (CNS) and play critical roles as regulators of CNS function, directors of neurodevelopment and responders to pathological processes. A wide range of neuroimmune factors are produced by CNS cells, primarily the glial cells, but the role of specific neuroimmune factors and their glial cell sources in CNS biology and pathology have yet to be fully elucidated. We have used transgenic mice that express elevated levels of a specific neuroimmune factor, the cytokine IL-6 or the chemokine CCL2, through genetic modification of astrocyte expression to identify targets of astrocyte produced IL-6 or CCL2 at the protein level. We found that in non-transgenic mice constitutive expression of IL-6 and CCL2 occurs in the two CNS regions studied, the hippocampus and cerebellum, as measured by ELISA. In the CCL2 transgenic mice elevated levels of CCL2 were evident in the hippocampus and cerebellum, whereas in the IL-6 transgenic mice, elevated levels of IL-6 were only evident in the cerebellum. Western blot analysis of the cellular and synaptic proteins in the hippocampus and cerebellum of the transgenic mice showed that the elevated levels of CCL2 or IL-6 resulted in alterations in the levels of specific proteins and that these actions differed for the two neuroimmune factors and for the two brain regions. These results are consistent with cell specific profiles of action for IL-6 and CCL2, actions that may be an important aspect of their respective roles in CNS physiology and pathophysiology.

## Introduction

Immune factors that play a critical role in the peripheral immune system, such as cytokines and chemokines, are now known to be produced within the CNS by cells of the CNS and to play important roles in normal CNS functions as well as pathological states (Gruol and Nelson, 1997; Glabinski and Ransohoff, 1999; Bajetto et al., 2001; Banisadr et al., 2005; Deverman and Patterson, 2009; Spooren et al., 2011; Erta et al., 2012; Arisi, 2014). Immune factors produced by CNS cells are referred to as neuroimmune factors to distinguish their origin from immune factors that are produced by cells of the peripheral immune system trafficketing through the CNS. The primary source of neuroimmune factors in the CNS is glial cells (astrocytes and microglia) (Kettenmann et al., 2011; Smith et al., 2012; Jensen et al., 2013; Choi et al., 2014). Depending on conditions, some neurons also produce neuroimmune factors (Tsakiri et al., 2008; Wei et al., 2013). Production of neuroimmune factors in the CNS is generally low, but increases significantly during adverse conditions such as injury and disease, when neuroimmune factors are thought to play important protective and/or repair roles. However, if the production of neuroimmune factors becomes dysregulated, the elevated levels may promote pathological processes rather than ameliorate the negative effects of adverse conditions.

CNS cells produce multiple neuroimmune factors, particularly during adverse conditions. The complexity of this situation makes it difficult to distinguish effects of individual neuroimmune factors and to identify target sites of action, information that is basic to an understanding of physiological and pathological roles and the development of new therapeutic strategies. Moreover, the cell source of neuroimmune factors under physiological or pathological conditions can be questionable, as multiple CNS cell types are capable of producing neuroimmune factors. To gain a clearer understanding of the actions and potential roles of neuroimmune factors in the CNS, we have focused on identifying the targets of individual neuroimmune factors when the initial source is CNS astrocytes. Astrocytes are closely associated with neurons and synapses and are known to participate in processes that are essential for normal CNS function, such as regulating synaptic transmission (Halassa et al., 2007; Bernardinelli et al., 2014) and neurodevelopment (Corty and Freeman, 2013). Neurons express receptors for neuroimmune factors, consistent with a role for these factors in astrocyte-neuron interactions. Moreover, astrocytes also express receptors for the same neuroimmune factors that they produce, providing a mechanism for feedback control of astrocyte function, and indirectly astrocyte regulation of neuronal function.

Two neuroimmune factors, the cytokine IL-6 and the chemokine CCL2, are particularly relevant to a number of pathological conditions. Elevated levels of these neuroimmune factors in the CNS parenchyma or cerebrospinal fluid (CSF) have been reported for a number of CNS disease states, and in particular, disease states associated with altered cognitive function. For example, elevated levels of IL-6 occur in the CNS or CSF of patients with clinical depression (Miller and O’Callaghan, 2005; Jones and Thomsen, 2013), active epilepsy (Billiau et al., 2007), Alzheimer’s disease (Brosseron et al., 2014), HIV infection (Gallo et al., 1989) and inflammatory neurological disorders (Wullschleger et al., 2013). Similarly, elevated levels of CCL2 occur in the CNS or CSF in HIV infection (Cinque et al., 1998; Kelder et al., 1998), multiple sclerosis (Conductier et al., 2010), Alzheimer’s disease (Galimberti et al., 2006; Westin et al., 2012), epilepsy (Wu et al., 2008), and psychiatric disorders (Stuart and Baune, 2014). Although it is unknown whether IL-6 and CCL2 contribute to the cause and/or consequences of disease states and symptoms, correlative evidence indicates a potential role in the etiology of some disease states. For example, increased levels of IL-6 were observed in the CNS of depressed patients and correlated with the severity of symptoms (Lindqvist et al., 2009). IL-6 levels were significantly higher in CSF of systemic lupus erythematosus (SLE) patients that showed psychiatric manifestations compared with SLE patients that did not show psychiatric manifestations (Hirohata and Miyamoto, 1990; Hirohata et al., 2009). IL-6 levels were significantly higher in the CSF of patients with cerebrovascular disease with dementia compared with patients with cerebrovascular disease without dementia (Wada-Isoe et al., 2004). Elevated levels of IL-6 were found in the CNS and CSF of autistic patients and correlated with increased levels IL-6 expression by astrocytes (Vargas et al., 2005; Wei et al., 2011). Similarly, increased levels of CCL2 were observed in the CSF of HIV-infected individuals and correlated with the level of viral load and severity of dementia (Kelder et al., 1998). In older Alzheimer’s patients, increased levels of CCL2 in the CSF correlated with cognitive deficits (Galimberti et al., 2006). CSF levels of CCL2 increase significantly with the age of patients with and without neuropsychiatric disease, suggesting that CCL2 plays an important role in the detrimental effects of aging on the CNS (Blasko et al., 2006). Experimental evidence also indicates a role of IL-6 in aging, and in particular, the negative effects of aging on cognitive function (Godbout and Johnson, 2004; Lekander et al., 2011; Burton and Johnson, 2012).

In addition to a role in pathological conditions, expression of IL-6 and CCL2 at low levels in the normal CNS suggest physiological roles for these neuroimmune factors. Studies in experimental animals support such a role for IL-6, but information on CCL2 is lacking. For example, the highest level of mRNA for both IL-6 and IL-6 receptors in the CNS occurs in the hippocampus, suggesting an important physiological role for IL-6 in the hippocampus (Gadient and Otten, 1994a,b; Gruol and Nelson, 1997). Consistent with this possibility, recent studies showed that astrocyte produced IL-6 provides homeostatic control of synaptic function in hippocampal neuronal circuits involved in memory and learning, essential cognitive functions that are disrupted in Alzheimer’s disease and other neurologic disorders (Balschun et al., 2004; Baier et al., 2009; del Rey et al., 2013). In these studies, animals implanted with electrodes and subjected to a stimulation paradigm that induced long-term potentiation (LTP) in the hippocampus showed dramatically upregulated IL-6 gene expression (Jankowsky et al., 2000; Balschun et al., 2004; del Rey et al., 2013). LTP is a form of synaptic plasticity characterized by an enhancement in the strength of synaptic transmission that is thought to be a key cellular mechanism underlying memory and learning. Studies using combined *in situ* hybridization and immunohistochemistry identified astrocytes as the source of the elevated levels of IL-6 in the LTP experiments (Jankowsky et al., 2000). Consistent with these studies, IL-6 gene expression was shown to be upregulated in behavioral experiments involving a hippocampal-dependent learning task (i.e., a spatial learning task) (del Rey et al., 2013). Behavioral studies of IL-6 deficient mice are also consistent with a physiological role for IL-6 in hippocampal function. For example, IL-6 deficient mice showed impaired memory in a behavioral test of hippocampal dependent memory (i.e., the Morris water maze), a result consistent with a regulatory role for IL-6 in memory management (Baier et al., 2009).

Studies involving exogenous application of IL-6 or CCL2 also support the idea that IL-6 and CCL2 can regulate neuronal function and behavior, actions that could play a role in the physiological or pathophysiological consequences of IL-6 or CCL2 expression in the CNS. For example, our studies of cultured hippocampus and cerebellum show that acute or chronic exposure to IL-6 or CCL2 can alter the physiological properties of neurons including neuronal excitability, synaptic transmission and glutamate receptor function (e.g., Qiu et al., 1998; Nelson et al., 2002, 2004; Gruol and Nelson, 2005; van Gassen et al., 2005). Similarly, studies of hippocampal slices acutely isolated from animals and studied *in vitro* showed that exogenous application of IL-6 reduced LTP (Li et al., 1997; Tancredi et al., 2000), while exogenous application of CCL2 to hippocampal slices increased excitability and synaptic transmission (Zhou et al., 2011). Exogenous application of CCL2 also increased neuronal excitability in striatal slices from mice (Guyon et al., 2009). In parallel studies, intranigral injections of CCL2 in mice increased locomotor activity, a result consistent with the excitatory actions of CCL2 in the slice studies (Guyon et al., 2009). Increasing the CNS levels of IL-6 also altered the behavior of mice as assessed by several different behavioral tests. For example, increasing levels of IL-6 in the CNS of mice by the use of the adenovirus expression system resulted in impaired cognitive ability and altered synaptic function (Wei et al., 2012).

Thus, there is a growing body of knowledge that supports physiological and/or pathological roles for IL-6 and CCL2 in the CNS. However, the exact role of these neuroimmune factors and mechanisms underlying their CNS actions are yet to be elucidated. Our goal in the current study was to determine if specific cellular and synaptic proteins are targets of IL-6 or CCL2 action, effects that could contribute to the mechanisms underlying the physiological or pathological actions of these neuroimmune factors. For these studies, we took advantage of two transgenic mouse models that express elevated levels of IL-6 or CCL2 in the CNS. In the transgenic mouse models, the elevated levels of IL-6 or CCL2 were accomplished by gene manipulation of astrocyte expression. Astrocytes are the most abundant cell type in the CNS (Heneka et al., 2010) and a primary producer of IL-6 and CCL2 in the normal CNS and during pathological conditions (Farina et al., 2007; Qin and Benveniste, 2012; Jensen et al., 2013; Choi et al., 2014). Expression of IL-6 or CCL2 in the astrocytes of the respective transgenic mice is under control of the promoter for GFAP, an astrocyte specific structural protein. Thus, the elevated production of these two neuroimmune factors is likely to involve similar if not identical pathways, and, at least initially, result in a similar spatial distribution of the secreted peptide. Moreover, the restricted expression in astrocytes enables identification of the initial source of the elevated levels of IL-6 or CCL2 in the transgenic CNS.

Studies of mice from the IL-6 and CCL2 transgenic lines indicate that the elevated astrocyte expression of the respective neuroimmune factor has neurological effects, although most effects were not prominent until later in the lifespan and differ for the two transgenic lines. The IL-6 transgenic (tg) mice and their non-transgenic (non-tg) littermates used in our study are heterozygote, low expressor mice from the 167 line (Campbell et al., 1993). Several studies have described the neurologic deficits of these mice (Chiang et al., 1994; Heyser et al., 1997; Boztug et al., 2002; Vallieres et al., 2002; Samland et al., 2003; Nelson et al., 2012). The IL-6 tg mice progressively develop tremor and ataxia by 6 months of age, indicative of cerebellar dysfunction, and infrequently, seizures (Campbell et al., 1993). In tests of a hippocampal-dependent behavior, avoidance learning, the IL-6 tg mice show progressive deficits that were not prominent until 12 months of age. Thus, at 3 months of age there was no difference in the ability of the IL-6 tg and non-tg mice to learn the avoidance response, whereas at 6 months of age and older the IL-6 tg mice progressively exhibited more errors in learning than the non-tg mice (Heyser et al., 1997). Functional alterations at earlier ages (at 2 months of age) were also demonstrated by increased susceptibility of the IL-6 tg mice to kainic acid and NMDA induced seizures (Samland et al., 2003) and enhanced synaptic transmission in the IL-6 tg hippocampus studied *in vitro* (Nelson et al., 2012). Interestingly, in transgenic mice that express elevated levels of IL-6 through neuronal expression, including expression in hippocampal pyramidal neurons and cerebellar Purkinje neurons, astrogliosis but no neurological deficits were observed (Fattori et al., 1995), suggesting that the source of IL-6 is an important factor in the neuronal consequences of IL-6 actions in the CNS.

Characterization of the CCL2-tg mice is more limited than for the IL-6 tg mice. However, in the heterozygote CCL2-tg mice used in our studies, no overt CNS pathology or neurological impairments were observed up to 7 months of age (Huang et al., 2002, 2005). At older ages, neurological impairments such as difficulty with righting reflex and limb weakness were observed (Huang et al., 2005). Behavioral tests at 2–3 months of age showed no significant difference between the CCL2-tg and CCL2 non-tg animals in rotarod performance, a test of cerebellar function, or in a behavior test of cued and contextual fear conditioning, which involves hippocampal and amygdala function (Bray et al., 2013). However, *in vitro* studies of hippocampal synaptic function at 2–3 months of age showed increased excitability in CA1 pyramidal neurons in CCL2-tg hippocampus, indicative of neuroadaptive changes due to the enhanced astrocyte expression of CCL2 (Bray et al., 2013).

IL-6 and CCL2 produce their biological effects by acting at specific membrane receptors. The IL-6 receptor is a transmembrane receptor that lacks a signal transduction element. Instead, IL-6 receptors utilize a common transmembrane signaling receptor referred to as gp130 (glycoprotein 130). When activated by IL-6, the IL-6 receptor associates with gp130 and induces dimerization with a second gp130 (Taga and Kishimoto, 1997). The gp130/gp130 homodimer can then activate JAK, STAT3, and/or MAPK signaling cascades, which leads to gene expression and other downstream actions. gp130 is widely distributed throughout the CNS, but IL-6 receptor distribution is more restricted. IL-6 can also produce biological effects by trans-signaling, which results from shedding of the membrane receptor to form a soluble receptor or alternative splicing of IL-6 receptor mRNA (Rose-John et al., 2006). The soluble receptor after binding IL-6 can interact with gp130 in IL-6 receptor expressing cells and in cells that normally do not express IL-6 receptor but do express gp130. Studies suggest that trans-signaling plays a central role in pathological actions of IL-6 in the CNS (Rose-John et al., 2006; Burton et al., 2011; Campbell et al., 2014). In contrast to IL-6, receptors for chemokines including CCL2 are G-protein coupled receptors (GPCRs) linked to Gi/Go. A variety of signal transduction pathways are activated by chemokine receptors including MAPK. These signal transduction pathways can ultimately lead to altered gene expression and downstream changes in protein levels. Therefore, modulation of protein levels could be one mechanism through which IL-6 and CCL2 participate in homeostatic control of neuronal and glial function, engage in protection and/or repair, and/or take part in pathological processes.

To identify targets (direct or indirect) of astrocyte produced IL-6 and CCL2 that are involved in the CNS actions of these neuroimmune factors, we compared protein levels in the hippocampus and cerebellum of IL-6 tg and CCL2-tg mice and their non-tg littermates. Both CNS regions play key roles in behavior. The hippocampus plays a key role in memory and learning, while the cerebellum plays a key role in coordinated movement. Results show that the levels of specific cellular and synaptic proteins are altered by elevated astrocyte expression of IL-6 and/or CCL2 and that these actions differed for the two neuroimmune factors in an age and CNS region dependent manner. These results are consistent with cell specific profiles of action for IL-6 and CCL2, actions that may be an important aspect of their respective roles in CNS physiology and pathophysiology.

## Materials and methods

### Animals

All animal procedures were performed in accordance with the Scripps Research Institute and the National Institutes of Health Guideline for the Care and Use of Laboratory Animals. Animal facilities and experimental protocols were in accordance with the Association for the Assessment and Accreditation of Laboratory Animal Care. Heterozygote IL-6 or CCL2 (previously known as monocyte chemoattractant protein-1 or MCP-1) transgenic mice and their non-transgenic littermates (as controls) were used for all studies. The IL-6 line was obtained from Dr. Ian Campbell of the University of Sydney and the CCL2 line was obtained from Dr. Richard Ransohoff of the Cleveland Clinic Foundation. Methods for the generation of the transgenic lines were previously published. Briefly, for the IL-6 line, full-length murine IL-6 cDNA was modified and inserted into the glial fibrillary acidic protein (GFAP) gene. The hybrid (transgene) DNA was subsequently microinjected into fertilized eggs of (C57BL/6J × SJL) F_1_ hybrid mice (Campbell et al., 1993). Transgene expression in astrocytes was documented by *in situ* hybridization studies and expression of the lacZ reporter gene as assessed by immunohistochemical detection of β-gal (Campbell et al., 1993; Vallieres et al., 2002). For the CCL2 line, the murine CCL2 gene was placed under control of the huGFAP promoter and a purified GFAP-CCL2 fusion gene fragment was injected into fertilized eggs of SWXJ (H-21^q,s^) mice (Huang et al., 2002). For both lines, transgenic offspring were identified by analysis of tail DNA using standard protocols. Cut tail tips from individual animals were obtained at weaning (21–28 days postnatal). Tail DNA was extracted using the Mouse Tail Quick Extraction Kit (Pioneer Inc., San Diego CA). Mice positive for the IL-6 or CCL2 transgene were identified by PCR. Samples were prepared for PCR using the HotStart Taq Master Mix with Loading Dye (Pioneer). Both lines are congenic and have been maintained for several years by breeding heterozygous transgenic mice with wildtype C57BL/6J mice.

### Protein assays

Protein samples for ELISA or Western blot assays were prepared from hippocampus and cerebellum of transgenic and non-transgenic mice using standard protocols. To obtain the tissue, mice were anesthetized with isoflurane and decapitated. The brains were rapidly removed and immersed in chilled artificial cerebral spinal fluid (ACSF). The ACSF composition was 130.0 mM NaCl, 3.5 mM KCl, 1.25 mM NaH_2_PO_4_, 24.0 mM NaHCO_3_, 0.2 mM CaCl_2_, 5.0 mM MgSO_4_, and 10.0 mM glucose (all chemicals from Sigma-Aldrich, St. Louis, MO). The ACSF was maintained on ice and was bubbled continuously with 95% O_2_/5% CO_2_ to provide oxygen and to stabilize pH at 7.2–7.4. The hippocampus and cerebellum were dissected from the brain, snap frozen on dry ice and stored at −80°C until use. In some studies, instead of immediately freezing the hippocampus, hippocampal slices were prepared using a protocol previously described (Nelson et al., 2011). Briefly, after cooling the brain in ACSF, the left and right hippocampi were removed from the brain and cut into 400 μm slices using a McIlwain tissue chopper (Mickle Laboratory Engineering Co. Ltd., Surrey, UK). Approximately six slices were obtained from each hippocampus. The slices were placed in two gas-fluid interface chambers maintained at ~33°C and were continuously superfused with oxygenated ACSF at rate of 0.55 ml/min to retain viability. After 2 h incubation, slices were pooled according to chamber, snap frozen and stored at −80°C.

Proteins were extracted from all tissue samples by sonication in cold lysis buffer containing 50 mM Tris-HCL, pH 7.5, 150 mM NaCl, 2 mM EDTA, 1% Triton X-100, 0.5% NP-40, a Complete Protease Inhibitor Cocktail Tablet (Roche Diagnostics, Mannheim, Germany), and a cocktail of phosphatase inhibitors (Na^+^ pyrophosphate, β-glycerophosphate, NaF, Na^+^ orthovanadate; all from Sigma-Aldrich). The samples were incubated on ice for 30 min, centrifuged at 13,860 g for 30 min at 4°C, and the supernatants were collected. Protein concentration in the supernatants was determined using the Bio-Rad Protein Assay Kit (Bio-Rad, Hercules, CA). Aliquots were stored at −80°C.

IL-6 and/or CCL2 levels in hippocampal and cerebellar protein samples were determined by ELISA using the Mouse IL-6 ELISA Ready-SET-Go! Kit or the Mouse CCL2 ELISA Ready-SET-Go! Kit, respectively (eBioscience, Inc., San Diego, CA) following manufacturer’s instructions. Levels of other proteins were determined by Western blot following previously published protocols (Nelson et al., 2012). Briefly, equal amounts of hippocampal or cerebellar protein samples were subjected to SDS-PAGE using 4–12% Novex NuPAGE Bis-Tris gels (Invitrogen Life Technologies, Grand Island, NY). Transgenic and non-transgenic protein samples were run on the same gel. Samples were run in duplicate. Proteins were transferred to Immobilon-P membranes (Millipore, Billerica, MA) and uniform transfer assessed by Ponceau S staining (Pierce, Rockford, IL). Membranes were washed and blocked in a 5% casein solution (Pierce). The membranes were incubated in primary antibody overnight (4°C), washed, and then incubated (room temperature) in secondary antibody coupled to horseradish peroxidase (HRP). Protein bands were visualized by chemiluminescence and quantified by densitometry measurements using NIH Image software.^1^ Membranes were stripped and reprobed for β-actin. To adjust for possible loading errors, the density of each band was normalized to the density of the band for β-actin in the same lane. Normalized data from transgenic mice were then normalized to the average normalized value for non-transgenic mice run on the same gel. In some studies to enable comparisons, hippocampal and cerebellar samples were run on the same ELISA and/or Western blot. Also, in some studies CCL2 and IL-6 samples were run on the same ELISA and/or Western blot. Data were combined according to mouse line, genotype and age of the animal and reported as mean ± SEM.

The following antibodies were used for Western blot studies: a monoclonal antibody to β-actin (#AC-15, 1:5000; Sigma, St. Louis, Missouri); a monoclonal antibody to GFAP (#MAB360; 1:10,000; Millipore); a monoclonal antibody raised against neuron specific enolase (#MAB314; 1:5000; Millipore); a mouse monoclonal antibody to glutamine synthetase (Glu syn) raised against a recombinant fragment corresponding to amino acids 274–374 of human glutamine synthetase (ab64613; 1:1000; abcam, Cambridge, MA); a purified rabbit polyclonal antibody raised against a peptide mapping at the carboxy terminus of C/EBP beta (C-19; 1:500; Santa Cruz Biotechnology, Santa Cruz, CA); a rabbit polyclonal antibody raised against a synthetic peptide to the C-terminus of rat GAD 65/67 (#AB1511; 1:1000; Millipore); a purified rabbit antibody to synapsin 1 (#51-5200; 1:5,000; Invitrogen; Syn 1); a purified rabbit polyclonal antibody raised against a synthetic peptide of the rat GluA1 subunit of the AMPA receptor conjugated to keyhole limpet hemocyanin (KLH; a protein carrier) with a cysteine added (#07-660; 1:500; Millipore); a purified goat polyclonal antibody raised against a peptide corresponding to an amino acid mapping the C-terminus of the human GluN1 subunit of the NMDA receptor (sc-1467; 1:500; Santa Cruz Biotechnology); a rabbit polyclonal antibody raised against p44/p42 MAPK (#61-7400; 1:5000, Zymed, Carlsbad, CA, USA); a purified rabbit polyclonal antibody raised against a synthetic phospho-peptide (KLH-coupled) corresponding to residues around Thr202/Tyr204 of human p44/p42 MAPK (#9101; 1:500; Cell Signaling Technologies, Danvers, MA; pp44/42 MAPK); a purified rabbit polyclonal antibody raised against a synthetic peptide (KLH-coupled) corresponding to the sequence of mouse STAT3 (AB#9132; 1:1000; Cell Signaling Technologies), a purified rabbit polyclonal antibody raised against a synthetic phospho-peptide (KLH-coupled) corresponding to the residues surrounding Tyr705 of mouse STAT3 (AB#9131; 1:1000; Cell Signaling Technologies).

### Statistical analyses

Statistical analyses of differences were performed using the unpaired *t*-test or ANOVA. Statistical significance was set at *p* < 0.05. For most studies, statistical comparisons were made between transgenic (IL-6 tg or CCL2-tg) vs. non-transgenic (IL-6 non-tg or CCL2 non-tg) values for hippocampus or cerebellum within the same age group and mouse line. For the IL-6 line results were obtained from: (a) mice 1–2 months of age: 25 IL-6 tg and 31 non-tg hippocampi (52 Western blots) and 9 IL-6 tg and 12 non-tg cerebella (73 Western blots); (b) mice 3–5 months of age: 42 IL-6 tg and 41 non-tg hippocampi (69 Western blots) and 9 IL-6 tg and 10 non-tg cerebella (17 Western blots); (c) mice 12 months of age: 10 IL-6 tg and 9 non-tg hippocampi (41 Western blots) and 11 IL-6 tg and 8 non-tg cerebella (21 Western blots). For the CCL2 line results were obtained from: (a) mice 1–2 months of age: 11 CCL2-tg and 11 non-tg hippocampi (34 Western blots) and 8 CCL2-tg and 9 non-tg cerebella (7 Western blots); (b) mice 3–5 months of age: 30 CCL2-tg and 23 non-tg hippocampi (56 Western blots) and 25 CCL2-tg and 26 non-tg cerebella (15 Western blots); and (c) mice 7–9 months of age: 26 CCL2-tg and 26 non-tg hippocampi (45 Western blots).

## Results

### IL-6 and CCL2 expression

**Figure 1:**
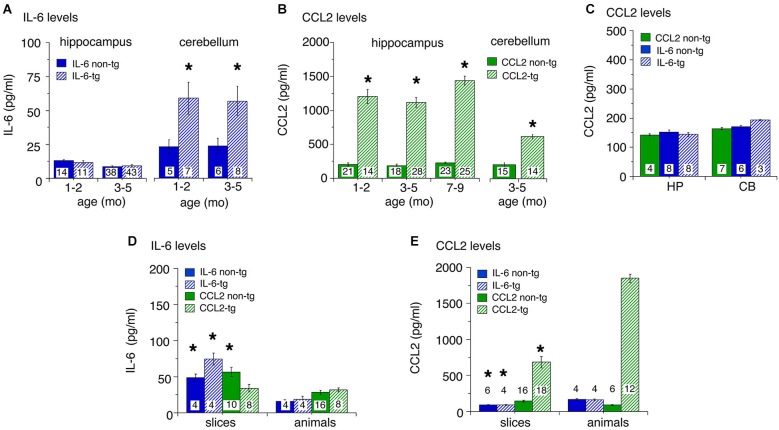
**Levels of IL-6 and CCL2 protein in transgenic and non-transgenic hippocampus and cerebellum determined by ELISA**. **(A)** IL-6 levels (mean ± SEM) in the hippocampus and cerebellum of IL-6 tg and non-tg mice at different ages. **(B)** CCL2 levels (mean ± SEM) in CCL2-tg and non-tg hippocampus and cerebellum at different ages. **(C)** CCL2 levels (mean ± SEM) in CCL2 non-tg hippocampus and cerebellum and IL-6 tg and non-tg hippocampus and cerebellum at 3–5 months of age. Numbers in bars are the number animals measured. * = statistically significant difference (*p* < 0.05, unpaired*t*-test) from non-tg of the same treatment group. **(D,E)** IL-6 **(D)** and CCL2 **(E)** levels in hippocampal samples with *in vitro* incubation (slices) or without (animals). For slices, numbers in bars are the number of samples measured (two samples/animal; left and right hippocampus). For animals, numbers in bars are the numbers of animals measured. * = statistically significant difference (*p* < 0.05, unpaired *t*-test) between slices and animal samples of the same genotype.

Levels of IL-6 and CCL2 in hippocampus and cerebellum of transgenic and non-transgenic mice at different ages were determined by ELISA. Results are shown in Figure 1. For the IL-6 line, low levels of IL-6 were observed in the hippocampus with no apparent genotypic difference (Figure 1A). IL-6 levels in the cerebellum were higher than in the hippocampus and a significant genotypic difference was observed (IL-6 tg > non-tg; Figure 1A). No prominent age-dependent differences were observed for levels of IL-6 in IL-6 tg and non-tg hippocampus and cerebellum. In contrast, levels of CCL2 were prominent in the CCL2-tg hippocampus and higher in the hippocampus than in the cerebellum. CCL2 levels showed a significant genotypic difference (CCL2-tg > non-tg) in both CNS regions (Figure 1B). No prominent age-dependent differences were observed for levels of CCL2 in CCL2-tg and non-tg hippocampus. CCL2 levels were also measured in the IL-6 tg and non-tg hippocampus and cerebellum and were comparable to that observed in the CCL2 non-tg hippocampus and cerebellum (Figure 1C). Similarly, IL-6 levels were measured in the CCL2-tg and non-tg hippocampus and cerebellum and were comparable to that observed in the IL-6 non-tg hippocampus and cerebellum (data not shown).

To determine if astrocytes constitutively secrete IL-6 or CCL2, an *in vitro* experimental protocol was used. In these studies, hippocampal slices were prepared from transgenic and non-transgenic mice and IL-6 or CCL2 levels were measured in the tissue after an *in vitro* incubation period. The rationale for these studies is that if the astrocytes in the CCL2-tg or IL-6 tg hippocampus constitutively secrete CCL2 or IL-6, respectively, tissue levels would decline during the incubation period, provided that the rate of synthesis did not match release. To identify such a change, tissue levels in the slices after incubation were compared to levels in hippocampal tissue that was directly snap frozen after removal from the animal. The protocol for the preparation and maintenance of the slices followed a standard protocol used for neurophysiological studies of hippocampal slices *in vitro* (e.g., Nelson et al., 2012). The slices were incubated for 2 h under physiological conditions with constant superfusion of oxygenated ACSF to maintain viability and remove secreted protein from the slices. The slices were then snap frozen for later protein assay. Results from ELISA measurements are shown in Figures 1D,E. Hippocampal samples prepared from tissue directly snap frozen after removal from the animal were measured in the same ELISAs. Levels of IL-6 in slices were generally higher than in the animal samples (Figure 1D), whereas the opposite was the case for CCL2 (Figure 1E). The most dramatic effect was a prominent reduction in the level of CCL2 in CCL2-tg hippocampal slices compared with hippocampal samples directly obtained from CCL2-tg animals. This result is consistent with constitutive astrocyte secretion of CCL2 (Figure 1E). Although constitutive secretion of IL-6 from astrocytes may have occurred, the higher levels of IL-6 in the hippocampal slices confounded assessment of secretion. The higher levels of IL-6 in the slices may have resulted from increased production, perhaps induced by the experimental manipulations. Alternatively, the higher levels could reflect reduced secretion, perhaps because IL-6 secretion from astrocytes requires a specific trigger.

Taken together, results from these studies show prominent differences in the levels of astrocyte produced IL-6 or CCL2 in the respective transgenic mice both within and across CNS regions. They also show that *in vitro* incubation differentially alters the levels of IL-6 and CCL2 in hippocampal tissue. These differences indicate that astrocyte production and/or trafficking varies for these two neuroimmune factors. In addition, these results show that increased expression of IL-6 does not trigger increased expression of CCL2 in the two CNS regions studied.

### Housekeeping proteins

**Figure 2:**
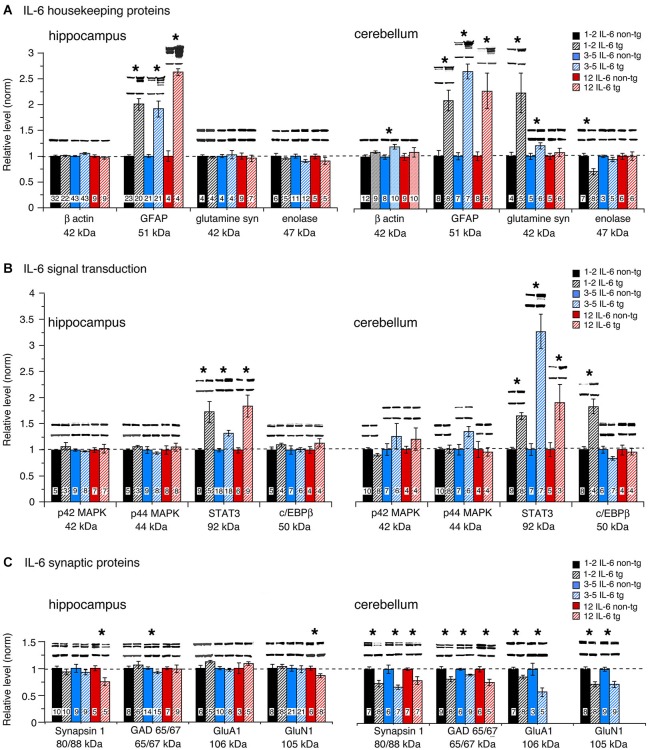
**Protein levels in hippocampus and cerebellum of IL-6 tg and non-tg mice at different ages determined by Western blot**. **(A)** Mean (±SEM) values for levels of housekeeping proteins. **(B)** Mean (±SEM) values for levels of signal transduction proteins. **(C)** Mean (±SEM) values for levels of synaptic proteins. Representative Western blots are shown above the respective bar. Top blot is the protein indicated for the graph; bottom blot is β actin in the same lane. Numbers in bars are the number of animals measured. * = statistically significant difference between IL-6 tg and non-tg for the age group.

**Figure 3:**
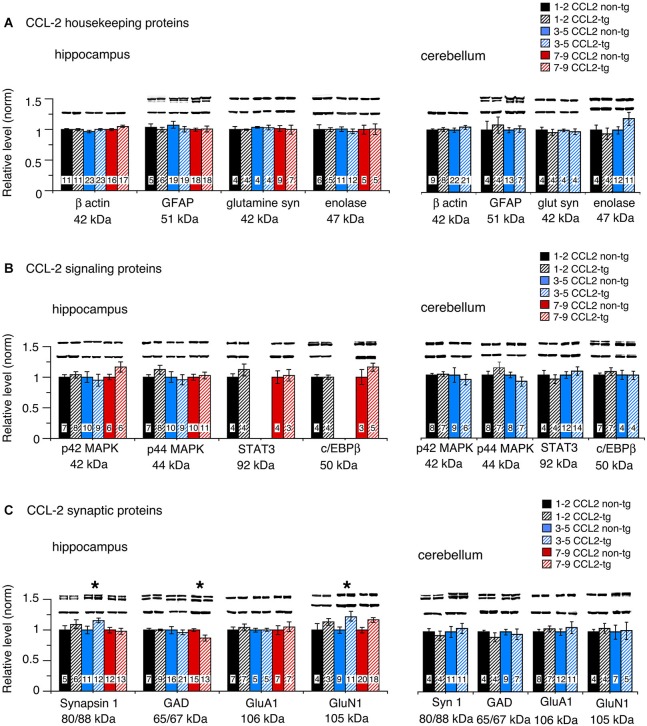
**Protein levels in hippocampus and cerebellum of CCL2-tg and non-tg mice at different ages determined by Western blot**. **(A)** Mean (±SEM) values for levels of housekeeping proteins. **(B)** Mean (±SEM) values for levels of signal transduction proteins. **(C)** Mean (±SEM) values for levels of synaptic proteins. Representative Western blots are shown above the respective bar. Top blot is the protein indicated for the graph; bottom blot is β actin in the same lane. Numbers in bars are the number of animals measured. * = statistically significant difference between CCL2-tg and non-tg for the age group.

To assess global effects of upregulated astrocyte expression of IL-6 or CCL2 in the hippocampus and cerebellum, several housekeeping proteins were measured by Western blot in tissue obtained from transgenic and non-transgenic mice at different ages. These proteins included β-actin, a cytosketal protein expressed in all cells, GFAP, an astrocyte specific cytosketal protein, glutamine synthetase, a protein important in astrocyte trafficking of glutamate, and neuron specific enolase, a specific neuronal protein. Results are shown in Figures 2, 3. In the IL-6 line, a significant genotypic difference (IL-6 tg > non-tg) was observed for GFAP levels in the hippocampus and cerebellum at all ages studied (Figure 2A). There was no significant genotypic difference in the levels of β-actin, glutamine synthetase and enolase in hippocampus at all ages studied. However, age-dependent genotypic differences were observed in the cerebellum. A prominent increase in the level of glutamine synthetase was observed at 1–2 months of age in the IL-6 tg cerebellum with a smaller increase at 3–5 months of age. A significant decrease in the level of enolase was also observed in the IL-6 tg cerebellum at 1–2 months of age. In addition, β-actin levels showed an increase in the IL-6 tg cerebellum at 3–5 months of age. No genotypic differences were observed in the IL-6 cerebellum at other ages studied. In contrast, in the CCL2 line, there were no genotypic differences in the levels of GFAP, β-actin, glutamine synthetase and enolase in the hippocampus or cerebellum at the ages studied (Figure 3A).

Taken together, these results show that increased expression of IL-6 in the IL-6 tg hippocampus and cerebellum, although not detected by ELISA in the hippocampus, has a pronounced effect on the expression of some hippocampal and cerebellar housekeeping proteins such as GFAP. Increased expression of CCL2, which can be detected by ELISA, has no apparent effect on levels of the same proteins. In addition, the absence of increased levels of GFAP in the CCL2-tg hippocampus and cerebellum indicates that CCL2 does not induce upregulated levels of IL-6 in these CNS regions of the CCL2-tg mice.

### Signal transduction proteins

The level of signal transduction proteins p42/44 MAPK, STAT3 and C/EBPβ were also measured by Western blot in the hippocampus and cerebellum of the transgenic and non-transgenic mice (Figures 2B, 3B). All three signal transduction proteins are known to be important regulators of normal neuronal and glial development and function (Sterneck and Johnson, 1998; Taubenfeld et al., 2001; Thomas and Huganir, 2004; Paquin et al., 2005; Ejarque-Ortiz et al., 2007; Dziennis and Alkayed, 2008; Kfoury and Kapatos, 2009; Cheng et al., 2013; Nicolas et al., 2013). For example, in the hippocampus, p42/44 MAPK and STAT3 play central roles in synaptic plasticity involved in memory and learning (Sweatt, 2001; Nicolas et al., 2012). Importantly, STAT3 and p42/44 MAPK are key players in IL-6 signal transduction and p42/44 MAPK is also involved in CCL2 signal transduction.

No significant genotypic differences in the levels of p42/44 MAPK were observed in the IL-6 hippocampus or cerebellum at the ages studied (Figure 2B). In contrast, a significant increase in STAT3 levels was observed in the IL-6 tg hippocampus and cerebellum at all ages studied (Figure 2B). C/EBPβ levels were prominently increased in the IL-6 tg cerebellum at 1–2 months of age, but not at other ages or in the IL-6 tg hippocampus (Figure 2B). No genotypic differences were observed in the hippocampus and cerebellum of the CCL2 line for p42/44 MAPK, STAT3 or C/EBPβ (Figure 3B).

Levels of the active form (i.e., phosphorylated form) of STAT3 (pSTAT3) were also examined in hippocampus and cerebellum from IL-6 tg and non-tg mice (3–5 months of age). Results showed that pSTAT3 levels were prominently increased in the IL-6 tg hippocampus (normalized values—tg/non-tg: IL-6 tg = 4.0 ± 0.9, *n* = 7; IL-6 non-tg = 1.0 ± 0.2, *n* = 7) and cerebellum (normalized values—tg/non-tg: IL-6 tg = 297 ± 102, *n* = 9; IL-6 non-tg = 1.0 ± 0.2, *n* = 7). Levels of the active form (i.e., phosphorylated form) of p42/44 MAPK (pp42/44 MAPK) were examined in hippocampus from IL-6 tg and non-tg mice (3–5 months of age) and in hippocampus from CCL2-tg and non-tg mice (3–9 months of age). The IL-6 tg hippocampus showed a 30% increase in pp42/44 MAPK relative to the non-tg hippocampus (normalized values—tg/non-tg: pp42 MAPK, IL-6 tg = 1.3 ± 0.1, *n* = 9; IL-6 non-tg = 1.0 ± 0.1, *n* = 8; pp44 MAPK, IL-6 tg = 1.3 ± 0.1, *n* = 9; IL-6 non-tg = 1.0 ± 0.1, *n* = 8). The CCL2-tg hippocampus showed a 30% decrease in pp44 MAPK but no change in pp42 MAPK relative to the non-tg hippocampus (normalized values—tg/non-tg: pp42 MAPK, CCL2-tg = 1.0 ± 0.1, *n* = 17; CCL2 non-tg = 1.0 ± 0.05, *n* = 19; pp44 MAPK, CCL2-tg = 0.7 ± 0.1, *n* = 10; CCL2 non-tg = 1.0 ± 0.1, *n* = 13).

### Synaptic proteins

Our previous studies showed that upregulated astrocyte expression of IL-6 or CCL2 in the respective transgenic mice produced neuroadaptive changes that altered synaptic function (Nelson et al., 2011, 2012; Bray et al., 2013). To determine if neuroadaptive changes occurred at the level of synaptic protein expression, we examined pre- and post-synaptic proteins by Western blot in hippocampus and cerebellum from transgenic and non-transgenic mice. Two presynaptic proteins were examined, Syn 1, a synaptic vesicle protein involved in transmitter release, and GAD65/67, the synthetic enzyme for the inhibitory transmitter GABA. Two postsynaptic proteins were also examined, GluA1, a subunit of the AMPA receptor, and GluN1, a subunit of the NMDA receptor. Both glutamate receptors, GluA1 and GluN1, are important mediators of excitatory synaptic transmission in hippocampal and cerebellar circuits.

Results show both age and region-dependent genotypic differences, the most pronounced effects occurring in the IL-6 tg cerebellum. In the IL-6 line, there was a small (~7%) but significant decrease in GAD65/67 in the IL-6 tg hippocampus at 3–5 months of age and a significant decrease in the levels of Syn 1 and GluN1 at 12 months of age (Figure 2C). Levels of Syn 1, GAD65/67, GluA1 and GluN1 were all significantly reduced in the IL-6 tg cerebellum at all ages studied. In the CCL2-tg hippocampus, levels of Syn 1 and GluN1 were significantly increased at 3–5 months of age, whereas the level of GAD 65/67 was significantly decreased at 7–9 months of age (Figure 3C). There was no significant genotypic difference in levels of Syn 1, GAD65/67, GluA1 and GluN1 in the CCL2-tg cerebellum at all ages studied (Figure 3C).

## Discussion

In the current study we measured the relative levels of cellular and synaptic proteins expressed in the hippocampus and cerebellum of transgenic mice that are genetically modified to produce elevated levels of IL-6 or CCL2 in CNS astrocytes. The goal of these studies was to identify CNS targets of the astrocyte produced IL-6 or CCL2, as evidenced by a change in the level of protein expression. Measurements were made at several ages in the lifespan of the animals, to reveal changes that could reflect developmental sensitivity and/or duration of exposure. Results show several changes in the level of neuronal and glial proteins in the hippocampus and cerebellum of the IL-6 tg mice but relatively few in the CCL2-tg mice (Table 1). Some changes were age and/or region specific. The ability of elevated levels of IL-6 or CCL2 to produce such changes could play a role in neurological, psychiatric or other disorders that show persistently elevated levels of IL-6 or CCL2 in the CNS. Moreover, IL-6 or CCL2 regulation of these targets could play a role in the normal physiological function of these CNS regions.

**Table 1:** **Changes in protein expression in hippocampus and cerebellum of IL-6 and CCL2 transgenic mice**.

	IL-6						CCL2					
	Hippocampus			Cerebellum			Hippocampus			Cerebellum		
Protein	1–2 months	3–5 months	12 months	1–2 months	3–5 months	12 months	1–2 months	3–5 months	7–9 months	1–2 months	3–5 months	7–9 months
β-actin	none	none	none	none	▲	none	none	none	none	none	none	nd
GFAP	▲	▲	▲	▲	▲	▲	none	none	none	none	none	nd
Glu syn	none	none	none	▲	▲	none	none	none	none	none	none	nd
Enolase	none	none	none	▼	none	none	none	none	none	none	none	nd
p42 MAPK	none	none	none	none	none	none	none	none	none	none	none	nd
p44 MAPK	none	none	none	none	none	none	none	none	none	none	none	nd
STAT3	▲	▲	▲	▲	▲	▲	none	nd	none	none	none	nd
C/EBPβ	none	none	none	▲	none	none	none	nd	none	none	none	nd
Synapsin 1	none	none	▼	▼	▼	▼	none	none	none	none	none	nd
GAD65/67	none	▼	none	▼	▼	▼	none	none	▼	none	none	nd
GluA1	none	none	none	▼	▼	nd	none	none	none	none	none	nd
GluN1	none	none	▼	▼	▼	nd	none	▲	none	none	none	nd

* for each group, tg is compared to non-tg; ▲ = tg > non-tg; ▼ = tg < non-tg; none = no significant difference between tg and non-tg; nd = not determined.

ELISA studies confirmed the presence of elevated levels of IL-6 and CCL2 in the hippocampus and/or cerebellum of the respective transgenic mice at all ages studied. Elevated levels were observed at the youngest age studied, 1–2 months of age, with no apparent age-dependent difference in the expression pattern at older ages. Animals at 1–2 months of age are considered to be adolescent/young adults, whereas the older ages (3–5 and 7–12) studied are considered to be adult ages. Astrocytes normally start to produce GFAP about 1 week postnatal with expression continuing throughout the lifetime of the animal. CNS expression of mRNA for IL-6 and CCL2 also begins during the postnatal period in normal animals (Gadient and Otten, 1994a; Pousset, 1994). CNS expression of IL-6 mRNA in the IL-6 transgenic mice was evident at 1 week postnatal and reached a maximum at 3 months of age (Chiang et al., 1994). GFAP mRNA expression, which is regulated by IL-6, and GFAP protein were also evident at 1 week postnatal but an increased level of GFAP in the IL-6 tg CNS was not prominent until about 1 month postnatal (Chiang et al., 1994). These results suggest that the consequences of elevated expression of IL-6 in the CNS are not prominent until about 1 month of age. CCL2 expression in CCL2-tg mice has not been studied at early ages but it is likely that the time course of expression is similar to that for IL-6, because the elevated levels of expression for both IL-6 and CCL2 is linked to GFAP expression in astrocytes (Brenner et al., 1994).

Elevated levels of IL-6 were not observed in the CCL2-tg hippocampus or cerebellum, indicating that elevated levels of CCL2 did not induce production of IL-6 in these CNS regions. Similarly, elevated levels of CCL2 were not observed in the IL-6 tg hippocampus and cerebellum, suggesting that elevated levels of IL-6 did not induce production of CCL2. These results are consistent with *in vitro* studies of cultured astrocytes involving exogenous application of IL-6 or CCL2 (Oh et al., 1999; Semple et al., 2010) and suggest that glial activation was not prominent in the transgenic mice. In both the transgenic and non-transgenic hippocampus and cerebellum, levels of CCL2 were considerably higher than levels of IL-6. This difference was also observed in CSF samples from human control subjects, the most common site for measurement of neuroimmune factors in humans. For example, IL-6 levels were reported to be ~1 pg/ml in the human CSF (Lindqvist et al., 2009) compared with ~840 pg/ml for CCL2 in human CSF (Janelidze et al., 2013).

Elevated levels of CCL2 were observed in the hippocampus and cerebellum of the CCL2-tg mice, but elevated levels of IL-6 were only observed in the cerebellum in the IL-6 tg mice. Moreover, IL-6 levels were higher in the cerebellum than in the hippocampus of the IL-6 tg mice, whereas in the CCL2 tg mice, CCL2 levels were higher in the hippocampus than the cerebellum. These regional difference may relate to differences in the production of these two neuroimmune factors by the Bergman glia, which are present in the cerebellum but not in the hippocampus. The Bergman glia express the highest level of IL-6 transgene in the IL-6 transgenic CNS (Campbell et al., 1993). The high levels of IL-6 in the IL-6 tg cerebellum may contribute to the damage and cell loss observed in this brain region at older ages, and consequently the ataxia characteristic of the older IL-6 tg mice (Campbell et al., 1993). A neurotoxic effect of high concentrations of IL-6 is consistent with our previous studies in a culture model, which showed that neurotoxicity was produced when cultured cerebellar granule neurons were exposed chronically to high concentrations of IL-6 in the culture media (e.g., 5–10 ng/ml) (Conroy et al., 2004).

Previous studies of rat brain showed higher levels IL-6 mRNA in the hippocampus than in the cerebellum (Gadient and Otten, 1994a), whereas our ELISA studies of non-tg mice showed higher levels of IL-6 protein in the cerebellum than in the hippocampus. Thus, there appears to be a mismatch between mRNA and protein levels. Because both cerebellar and hippocampal samples were run on the same ELISA, it is unlikely that technical difficulties account for this difference. Assuming that this apparent mismatch is not due to an across species comparison, it is likely that brain region specific post-translational events (e.g., post-translational regulation) are responsible for the mismatch between mRNA and protein levels observed in our studies, as has been shown for other proteins in other systems (Shebl et al., 2010; Khositseth et al., 2011; Vogel and Marcotte, 2012).

Although elevated levels of IL-6 were not observed in the IL-6 tg hippocampus, evidence for increased expression of IL-6 was provided by our Western blot results showing elevated levels GFAP, STAT3 and pSTAT3 in both hippocampus and cerebellum. GFAP levels are known to be regulated by IL-6 through a signal transduction pathway involving STAT3 (Heinrich et al., 1998; Herrmann et al., 2008; Shu et al., 2011). Therefore, the increased levels of GFAP, STAT3 and pSTAT3 provide evidence of increased production of IL-6. The differences in the ability to detect elevated levels of IL-6 vs. CCL2 in the respective transgenic tissue suggests that astrocyte trafficking differs for IL-6 and CCL2 and imply that IL-6 is likely to be released after production, whereas CCL2 can be released and/or stored. Studies of astrocytes cultured from the CNS of IL-6 tg or CCL2-tg mice demonstrated the ability of astrocytes to secrete IL-6 (Campbell et al., 1993) or CCL2 (Huang et al., 2002), respectively. For example, IL-6 bioactivity in the supernatant of cultured astrocytes obtained from the CNS of IL-6 tg mice was approximately 150 pg/ml compared with 5 pg/ml for IL-6 non-tg astrocytes (Campbell et al., 1993). CCL2 levels in the supernatant of cultured astrocytes obtained from the CNS of CCL2-tg mice was approximately 3500 pg/ml compared with 1 pg/ml for astrocytes from the CCL2 non-tg mice (Huang et al., 2002).

Differences in the levels of cellular and synaptic protein measured by Western blot between the IL-6 tg and IL-6 non-tg mice were observed for both the hippocampus and cerebellum. However, the cerebellum showed more prominent effects of IL-6 than the hippocampus, presumably due to the higher level of expression of IL-6 in the cerebellum. Differences in cell types that comprise the two regions could also be a contributing factor. The proteins most affected by the elevated levels of IL-6 in both CNS regions were GFAP, STAT3 and pSTAT3, which were all increased. GFAP is restricted to astrocytes, whereas STAT3 is expressed by both neurons and glia, as are IL-6 receptors (Schöbitz et al., 1992; Planas et al., 1997; Nelson et al., 1999; Murata et al., 2000). Because our studies used whole hippocampus and cerebellum, we were unable to identify the cell types or subcellular compartments that showed the increased STAT3 and pSTAT3 levels in these CNS regions. In previous studies of the IL-6 tg cerebellum, pSTAT3 was detected primarily in the nucleus of glial cells (Sanz et al., 2008), suggesting that glial cells are the primary cell type responding to the chronic expression of IL-6 in the IL-6 tg cerebellum. However, the primary site of neuronal effects may be at non-nuclear sites. Both STAT3 and pSTAT3 have been shown to be localized to synaptic sites in the hippocampus and cortex (Murata et al., 2000) and, independent of nuclear localization, to play a role in long-term depression, a form of synaptic plasticity essential for normal CNS function (Nicolas et al., 2012). LTP, another form of synaptic plasticity, has been shown to be regulated by IL-6 in the hippocampus and to involve glial release of the IL-6, which presumably acts at neuronal IL-6 receptors (Li et al., 1997; Jankowsky et al., 2000; Tancredi et al., 2000; Balschun et al., 2004). IL-6 signaling through IL-6 receptors involving both STAT3 and/or p42/44 MAPK has been demonstrated in neuronal cells (Schumann et al., 1999; Park et al., 2012; Fang et al., 2013). Thus, the elevated levels of STAT3 and/or pSTAT3 could occur primarily at synaptic or other subcellular sites in neurons in the IL-6 tg mice, a possibility that will require further investigation.

In addition to GFAP, another glial protein was increased in the IL-6 tg cerebellum, glutamine synthetase. This protein was not elevated in the IL-6 tg hippocampus and was only prominently elevated at 1–2 months of age in the IL-6 tg cerebellum. Glutamine synthetase plays a central role in the trafficking of glutamate, the primary excitatory transmitter in the CNS and a precursor for the inhibitory transmitter GABA (Mathews and Diamond, 2003; Albrecht et al., 2010; Tani et al., 2014). Glutamate that is released at excitatory synapses is taken up by astrocytes, converted to glutamine by glutamine synthetase and the glutamine is then released to the environment for neuronal uptake and processing. Glutamine synthetase is an important player in the neuroprotective role of astrocytes against glutamate toxicity (Zou et al., 2010; Zhang et al., 2011; Tani et al., 2014). An increased level of extracellular glutamate, which could lead to glutamate toxicity, induces an upregulation of glutamine synthetase (Lehmann et al., 2009). Thus, the increased levels of glutamine synthetase in the IL-6 tg cerebellum may play a protective role at early ages. Neuronal toxicity is known to occur in the IL-6 transgenic hippocampus and cerebellum, but does not become prominent until older ages (Heyser et al., 1997).

The transcription factor C/EBPβ was also elevated in the cerebellum and not the hippocampus of the IL-6 tg mice and only at 1–2 month of age. C/EBPβ is expressed prominently in neurons but also by glial in the CNS (Ejarque-Ortiz et al., 2007; Kfoury and Kapatos, 2009; Peña-Altamira et al., 2014). C/EBPβ regulates a host of neuronal genes (Kfoury and Kapatos, 2009) and its upregulation may be a contributing factor to the lower level of enolase in the IL-6 tg cerebellum at 1–2 months of age. In addition to enolase, several other neuronal proteins were reduced in the cerebellum at 1–2 months of age including Syn 1, GAD 65/67, GluA1 and GluN1, which are all synaptic proteins. However, the levels of these proteins were reduced at older ages, whereas the reduced level of enolase and increased level of C/EBPβ only occurred at 1–2 months of age. Whether or not the correlation between levels of enolase and C/EBPβ has a causative aspect will require further study. With respect to the decreased levels of synaptic proteins in the cerebellum, it seems unlikely that this decrease reflects neuronal loss, at least at 1–2 months of age when neuronal death is not prominent in the IL-6 tg cerebellum. Moreover, at older ages housekeeping and signal transduction proteins did not show a decrease, as would be expected if prominent cell loss occurred in the IL-6 tg cerebellum. One possible explanation is that the decreased levels of synaptic proteins reflect reduced axon or synapse formation that occurred during cerebellar development. Our previous studies using a culture model system showed that the level of α-internexin, a major neurofilament expressed in axons of cerebellar granule neurons (Chien et al., 1996), was reduced in granule neuron cultures exposed to elevated levels of IL-6 during development (Conroy et al., 2004). α-internexin has been proposed to play a role in neuronal maturation and axon stability (Chien et al., 1996) and in the development and organization of postsynaptic densities (Suzuki et al., 1997). Further studies will be necessary to determine if the changes in protein levels observed in the current study correlate with changes in cell structure or cell number.

In the IL-6 tg hippocampus, there were no genotypic differences for the levels of β-actin, glutamine synthetase, p42/44 MAPK, and C/EBPβ Based on the high abundance of these proteins in the hippocampus, it seems unlikely that the elevated levels of IL-6 resulted in prominent cell death, at least at the younger ages studied. This interpretation is consistent with studies using fluoro-jade staining for degenerating neurons in 3–4 month old IL-6 tg and non-tg hippocampus, which revealed no evidence of cell toxicity (Vallieres et al., 2002). Gross histological analysis of cresyl violet and TUNEL stained IL-6 tg and non-tg hippocampus also revealed no evidence of prominent cell loss at 2–6 months of age (Samland et al., 2003). The levels of Syn 1 and GluN1 were reduced in the IL-6 tg hippocampus but only at the oldest age studied (12 months of age). Decreased levels of Syn 1 in the IL-6 tg hippocampus at 12 months of age was also observed in a previous study (Heyser et al., 1997). The reduction in Syn 1 and GluN1 at the older age could reflect synaptic damage and/or neuronal loss, changes that could be important contributors to the deficits observed in studies of hippocampal-dependent behavior (Heyser et al., 1997).

In contrast to the IL-6 tg mice, in the CCL2-tg mice there was little evidence of genotypic differences in the level of cellular proteins for the hippocampus and no genotypic differences for the cerebellum. In the CCL2 hippocampus, a modest but significant increase in the levels of Syn 1 and GluR1 was observed at 3–5 months of age, a genotypic difference that was opposite to the reduced levels of these proteins observed in the IL-6 hippocampus at 12 months of age. However, a small but significant decrease in GAD65/67 was observed in both the CCL2-tg and IL-6 tg hippocampus, although for different age groups. Thus, IL-6 and CCL2 appear to directly or indirectly target some of the same synaptic proteins, effects that could have bearings relative to conditions where both neuroimmune factors are elevated in the CNS. There was no evidence of cell loss in the CCL2-tg mice, as evidenced by the relative lack of genotypic differences.

Taken together, results from our studies show specific profiles of action of IL-6 and CCL2 in the respective transgenic mice, with the most pronounced effects occurring in the cerebellum of the IL-6 tg mice. The pronounced changes in the cerebellum may be an important factor in the ataxia characteristic of the IL-6 tg mice. Although CCL2 did not appear to prominently affect the level of protein expression in the CCL2-tg mice, our previous studies showed that the persistently elevated levels of CCL2 in the hippocampus of the CCL2-tg mice produced neuroadaptive changes that altered aspects of synaptic function and sensitivity to alcohol (Nelson et al., 2011; Bray et al., 2013). Synaptic function was also altered in our studies of the hippocampus of IL-6 tg mice (Nelson et al., 2011), but the effects differed from that observed in the CCL2-tg mice. Thus, in the CCL2-tg hippocampus, somatic excitability was altered (increased), whereas in the IL-6 tg hippocampus, the dendritic synaptic response was altered (increased). The mechanisms mediating these neuroadaptive effects on synaptic function remain to be resolved but may involve some of the changes in protein expression observed in the current studies.

## Author contributions

Donna L. Gruol and Jennifer G. Bray designed the studies and wrote the paper. Donna L. Gruol, Khanh Vo and Jennifer G. Bray managed the mouse colony. Jennifer G. Bray performed the dissections. Donna L. Gruol and Khanh Vo carried out the biochemical analyses.

## Conflict of interest statement

The authors declare that the research was conducted in the absence of any commercial or financial relationships that could be construed as a potential conflict of interest.

## Footnotes

^1^http://rsb.info.nih.gov/nih-image/
